# Timing of external cephalic version at term: A cohort study

**DOI:** 10.1097/MD.0000000000050569

**Published:** 2026-09-11

**Authors:** Fayrooz Yossef, Shlomi Sagi, Rami Sammour, Inna Bleicher

**Affiliations:** a Department of Obstetrics and Gynecology, Bnai Zion Medical Center, Haifa, Israel; b Rappaport Faculty of Medicine, Technion Israel Institute of Technology, Haifa, Israel.

**Keywords:** breech presentation, cesarean delivery, fetal malpresentation, gestational age, neonatal outcomes

## Abstract

In patients with breech presentation at term, the study compared external cephalic version (ECV) success and cesarean delivery (CD) rates between early and late ECV. Although ECV is commonly used to reduce CD in breech presentation, the timing of ECV within the term period remains unclear. This was a retrospective cohort study at a university-affiliated medical center. We included patients with a singleton breech presentation at term who were eligible for ECV between January 2021 and March 2023. Data were collected from electronic medical records. Patients were categorized into 2 groups: early ECV (performed at ≤37 + 2 weeks) and late ECV (performed at ≥37 + 3 weeks). The primary outcome was ECV success. Secondary outcomes included mode of delivery and neonatal outcomes. Among 89 patients who underwent an ECV attempt (out of 240 eligible patients), 37 had early ECV and 52 had late ECV. Baseline characteristics were generally similar between groups. The late ECV group had a higher ECV success rate (83% vs 59%; odds ratio, 3.26; 95% confidence interval, 1.25–8.91) and a lower CD rate compared with the early ECV group (35% vs 59%; odds ratio, 0.38; 95% confidence interval, 0.16–0.91) in unadjusted analysis. ECV performed at ≥37 + 3 weeks was associated with higher ECV success and lower CD rates compared with earlier attempts. These findings suggest that the timing of ECV within the term period may be associated with ECV outcomes. Prospective studies are needed to confirm these findings.

## 1. Introduction

Approximately 3% to 4% of pregnancies at term involve a fetus in breech presentation, making the optimal mode of birth difficult to determine. The decision depends on multiple factors, including complication rates, patient preferences, and the risks and benefits of each approach.^[1,2]^ The Term Breech Trial demonstrated that planned cesarean delivery (CD) significantly reduces the risk of neonatal mortality and severe morbidity compared with planned vaginal birth in term breech pregnancies, leading to a global shift toward CD for breech presentation. This has been supported by more recent evidence.^[3]^ However, CD carries its own risks, including greater maternal morbidity, longer recovery, and complications in future pregnancies.^[4]^

External cephalic version (ECV) is therefore commonly offered as an alternative, allowing a trial of labor if successful.^[5]^ Despite the established benefits of ECV, the optimal timing within the term period remains unclear. Preterm ECV between 34 and 36 weeks has been associated with higher rates of cephalic presentation at birth, but also a higher risk of preterm birth, without a significant reduction in the overall CD rate compared with later attempts.^[6]^

Although prior studies have investigated the effectiveness of preterm versus term ECV, limited research has specifically compared early-term and late-term ECV within the term gestational window. This gap in evidence limits clear clinical guidance. Current guidelines generally advocate for ECV at or beyond 37 weeks, but the ideal timing within this range remains a subject of ongoing debate.

In our institution, ECV attempts are initiated at 37 + 0 weeks, resulting in a natural distribution of early and late ECV attempts.

This study aimed to examine whether the timing of ECV within the term gestational period is associated with ECV success and mode of delivery. We hypothesized that late-term ECV (≥37 + 3 weeks) would be associated with higher ECV success rates and lower CD rates.

## 2. Materials and methods

### 2.1. Study design and setting

This was a retrospective cohort study conducted at a single university-affiliated medical center between January 2021 and March 2023, with approximately 3600 annual deliveries. The hospital serves a diverse population, including urban and rural areas with varying socioeconomic backgrounds. In cases of unsuccessful ECV, patients were offered either a planned CD at our institution or referral to another hospital for a trial of vaginal breech birth. This study was approved by the institutional review board.

### 2.2. Study participants

Eligible patients with singleton breech presentation at term were identified. The analytic cohort included those who underwent an ECV attempt at our institution during the study period. Patients who had an early ECV (37 + 2 weeks or earlier) were compared with patients who had late ECV (37 + 3 weeks or later). We selected 37 + 2 weeks as the threshold for early ECV because patients are routinely scheduled for their first available ECV slot within 48 hours of reaching 37 + 0 weeks. Therefore, ≤37 + 2 weeks captures those offered an early-slot attempt, whereas ≥37 + 3 weeks represents patients who present after that window. Data were retrieved from electronic medical records (EMRs).

Inclusion criteria were patients with breech presentation, gestational age ≥37 weeks, and no contraindication for ECV attempt or vaginal birth. Patients were excluded if ECV date data were missing, if ECV was performed at another institution, or if delivery occurred at another institution following ECV. In addition, ECV was not performed in some patients due to labor onset, non-reassuring fetal heart rate, or suspected nuchal cord.

### 2.3. Variables

Baseline characteristics included maternal age, parity, body mass index, smoking status, prior CD, and gestational age at ECV. Labor and delivery variables included mode of birth, indications for labor induction, gestational age at delivery, ECV-to-delivery interval, timing of membrane rupture to delivery, perineal lacerations, and length of hospital stay. Neonatal data included birth weight, Apgar score at 5 minutes, and neonatal intensive care unit admission.

The primary outcome was ECV success. Secondary outcomes included mode of delivery, induction of labor method, ECV-to-delivery interval, and neonatal outcomes.

### 2.4. Data sources

Data were extracted from the EMRs retrospectively. Labor and delivery data are recorded as part of routine medical care and documented in real time by clinicians and midwives from admission until discharge.

### 2.5. Bias

The retrospective nature of this study has inherent biases, including reliance on EMRs, which may contain missing or incomplete data such as the date and time of ECV. Selection bias occurred because the analysis included only patients who underwent ECV at our institution and delivered there, which can limit the generalizability of the findings. In addition, confounding is possible from measured and unmeasured factors.

### 2.6. Statistical analysis

Continuous variables are presented as medians with interquartile ranges, and categorical variables as counts and percentages. Between-group comparisons were performed using the Mann–Whitney *U* test for continuous variables and Fisher’s exact test for categorical variables. Unadjusted odds ratios (ORs) with 95% confidence intervals (CIs) were calculated for selected binary outcomes. The study size was determined by the number of eligible ECV attempts during the study period. Exploratory analyses were performed for selected delivery outcomes. Statistical analyses were performed using R software (version 4.2.1; R Foundation for Statistical Computing, Vienna, Austria).

## 3. Results

**Figure 1.**
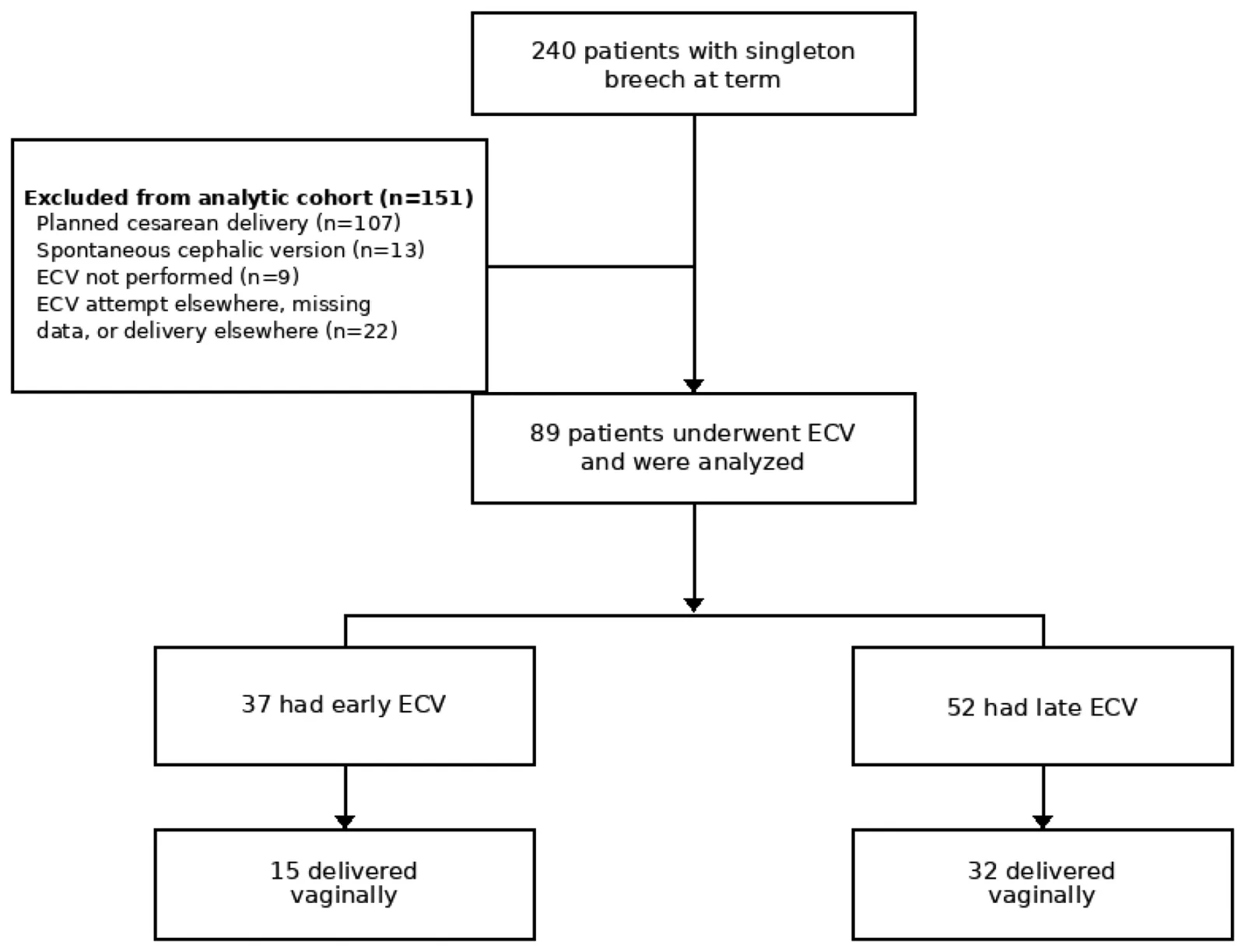
Study population and patient flow. ECV = external cephalic version.

A total of 240 patients with a singleton breech presentation were identified (Fig. 1). Of these, 151 were excluded from the analytic cohort (107 had a planned CD, 13 had spontaneous cephalic version, 9 did not undergo ECV, 10 had missing data, and 2 underwent ECV elsewhere). Finally, 89 patients had an ECV attempt and were included in the analysis, 37 in the early ECV group and 52 in the late ECV group.

Baseline demographic characteristics, including maternal age, parity, and body mass index, were similar between the groups (Table 1). The late ECV group had a higher ECV success rate (83% vs 59%; OR, 3.26; 95% CI, 1.25–8.91) and a lower CD rate compared with the early ECV group (35% vs 59%; OR, 0.38; 95% CI, 0.16–0.91).

**Table 1:** Patient characteristics.

	Early ECV (N = 37)*	Late ECV (N = 52)*	*P*-value
Maternal age (yr)	32.0 (29.0, 34.0)	32.0 (29.0, 36.0)	.6
Gestational age at ECV (wk)	37.1 (37.0, 37.3)	38.0 (37.6, 38.7)	<.001
BMI (kg/m^2^)	27.1 (24.5, 31.3)	29.1 (26.3, 31.4)	.7
Smoking	0 (0%)	1 (2.3%)	>.9
Nulliparous	17 (46%)	14 (27%)	.066
Prior cesarean delivery	2 (5.4%)	4 (7.7%)	.7

Gestational age is represented as weeks with 1 decimal place (e.g., 37 weeks 4 days = 37.6).

BMI = body mass index, ECV = external cephalic version.

* Data are presented as median (interquartile range) or number (%).

Further subanalysis showed that planned CD rates were lower in the late ECV group (19% vs 31%; unadjusted OR, 0.35; 95% CI, 0.14–0.86), while emergent CD rates were comparable between groups (7% vs 7%; unadjusted OR, 1.14; 95% CI, 0.28–4.57). The rate of vacuum-assisted deliveries and other maternal and neonatal outcomes were similar between groups (Table 2).

**Table 2:** Primary and secondary outcomes.

	Early ECV (N = 37)*	Late ECV (N = 52)*	*P*-value
Primary outcome			
ECV success	22 (59%)	43 (83%)	**.017**
Secondary outcomes			
Mode of delivery			
Vaginal delivery	15 (41%)	32 (62%)	
Vacuum-assisted delivery	0 (0%)	2 (3.8%)	>.9
Cesarean delivery	22 (59%)	18 (35%)	**.032**
Planned	11 (30%)	10 (19%)	.2
Emergency	3 (8.1%)	4 (7.7%)	>.9
Labor induction method			
Prostaglandin (misoprostol/dinoprostone)	5 (15%)	10 (20%)	.6
Double balloon catheter	3 (9.1%)	2 (3.9%)	.3
Oxytocin	2 (6.1%)	5 (9.8%)	.5
Other maternal outcomes			
Gestational age at delivery (wk)	39.1 (38.8, 39.8)	39.4 (38.8, 40.4)	.2
ECV-to-delivery interval (d)	13 (6, 15)	7 (2, 16)	.072
Membrane rupture to delivery (min)	26 (1, 263)	156 (2, 400)	.12
Perineal laceration	6 (23%)	18 (45%)	.075
Length of hospital stay (d)	3 (2, 3)	3 (2, 3)	.3
Neonatal outcomes			
Birth weight (g)	3360 (3220, 3645)	3305 (3020, 3730)	.5
Apgar score < 7 at 5 min	0 (0%)	1 (4.5%)	1
NICU admission	1 (2.7%)	2 (3.8%)	.8

Bold values indicate statistically significant differences (*P* < .05).

ECV = external cephalic version, NICU = neonatal intensive care unit.

* Data are presented as median (interquartile range) or number (%).

## 4. Discussion

Our findings suggest that late ECV (≥37 + 3 weeks) is associated with higher success rates and lower CD rates compared with early ECV. Further subanalysis showed that the difference in CD rates was explained by fewer planned procedures in the late group, while emergent CD rates did not differ, supporting the safety of later attempts.

These results are consistent with those of Kabiri et al, who similarly reported increased success rates for ECV performed later in gestation and decreased cesarean deliveries when ECV was done closer to term. However, our findings differ from those of Cahan et al, who observed no significant differences in CD rates related to the timing of ECV.^[7,8]^ An important methodological difference is that both prior studies defined timing based on the interval between ECV and delivery (e.g., <96 hours vs >96 hours) rather than using a defined gestational age window.

Our late-group success rate of 83% is in line with rates reported in the recent literature, which generally range from 50% to over 80%, depending on factors such as parity and gestational age.^[9,10]^ Notably, none of these studies specifically compared outcomes across defined gestational age windows within the term period, which limits direct comparison and highlights the gap our study aims to address.

Importantly, no significant differences were observed between the groups in neonatal outcomes, including birth weight and neonatal intensive care unit admissions. This is consistent with the broader literature supporting the safety of ECV at term, which has reported no serious fetal complications even in higher-risk populations, such as women with a prior CD.^[11,12]^ These findings suggest that while the timing of ECV may influence the mode of birth, it does not appear to negatively impact neonatal health.

This study has several limitations. First, the sample size was small, which limits statistical power and the ability to draw firm conclusions. Second, the retrospective design is subject to selection bias and incomplete documentation. Third, the findings may not be generalizable to other settings with different patient populations or ECV practices. Finally, nulliparity was more common in the early ECV group, which may have influenced the observed results.

Given these limitations, our results should be interpreted with caution. Further studies with larger sample sizes and, ideally, prospective designs are needed to confirm whether gestational age at ECV within the term window is a meaningful determinant of outcomes.

## 5. Conclusion

Our study suggests that performing ECV at ≥37 + 3 weeks may result in higher success rates and lower CD rates compared with earlier attempts, without compromising neonatal safety. However, given the study’s limitations, these findings should be interpreted with caution, and further studies with larger sample sizes are required to confirm these observations.

## Author contributions

**Conceptualization:** Fayrooz Yossef.

**Data curation:** Fayrooz Yossef.

**Methodology:** Inna Bleicher.

**Supervision:** Shlomi Sagi, Inna Bleicher.

**Writing – original draft:** Fayrooz Yossef, Inna Bleicher.

**Writing – review & editing:** Fayrooz Yossef, Shlomi Sagi, Rami Sammour, Inna Bleicher.
